# Development of Aspergillosis in a cohort of non-neutropenic, non-transplant patients colonised by *Aspergillus* spp

**DOI:** 10.1186/s12879-016-2143-5

**Published:** 2017-01-06

**Authors:** José Barberán, Francisco-Javier García-Pérez, Victoria Villena, Alberto Fernández-Villar, Eduardo Malmierca, Cristina Salas, María-José Giménez, Juan-José Granizo, Lorenzo Aguilar, on behalf of the working group on Infectious Diseases from the Spanish Society of Internal Medicine

**Affiliations:** 1 Internal Medicine Dpt., Hospital Universitario Monteprincipe, Universidad San Pablo-CEU, Boadilla del Monte, Avda. Monteprincipe 25, 28660 Boadilla del Monte, Madrid Spain; 2 Pneumology Dpt., Hospital Universitario de la Princesa, calle Diego de León 62, 28006 Madrid, Spain; 3 Pneumology Dpt., Hospital Universitario 12 de Octubre, Avda. de Córdoba s/n, 28041 Madrid, Spain; 4 Pneumology Dpt., Complexo Hospitalario Universitario de Vigo, Clara Campoamor 341, 36204 Vigo, Spain; 5 Internal Medicine Dpt., Hospital Universitario Infanta Sofia, Paseo de Europa 34, 28703 San Sebastián de los Reyes, Madrid Spain; 6 Internal Medicine Dpt., Hospital Universitario Marqués de Valdecilla, Avda. Valdecilla s/n, 39008 Santander, Spain; 7 PRISM-AG, calle Don Ramon de la Cruz 72, 28006 Madrid, Spain; 8 Preventive Medicine, Hospital Universitario Infanta Cristina, Avda. 9 de Junio 2, 28981 Parla, Madrid Spain

**Keywords:** Aspergillosis, Colonization, COPD, *Aspergillus*, Incubation period

## Abstract

**Background:**

A previous study explored factors discriminating colonization and true infection among non-transplant, non-neutropenic patients with repeated *Aspergillus* spp. isolation from lower respiratory samples. The present study explored the evolution of patients with *Aspergillus* colonization in that study to determine the percentage of cases progressing to aspergillosis and time to development.

**Methods:**

Clinical records were retrospectively reviewed (for each patient from his end date in the past study) and data from all respiratory processes suffered by patients up to April 2015 were recorded. Comparisons of variables were performed between colonized patients that developed aspergillosis and those that did not. A Kaplan-Meier curve was used to describe time to development of aspergillosis in chronic obstructive pulmonary disease (COPD) patients for II-IV stages of the Global Initiative for Chronic Obstructive Lung Disease (GOLD) classification.

**Results:**

Sixty seven colonized patients were followed, 12 of them (17.9%) developed aspergillosis. Diagnoses included six tracheobronchitis (4 invasive, 2 simple tracheobronchitis), four pulmonary disease (2 invasive pulmonary aspergillosis, 2 chronic pulmonary aspergillosis), one allergic bronchopulmonary aspergillosis and one pulmonary aspergilloma. Up to 47 (70.4%) of the study patients presented COPD. Among patients developing aspergillosis COPD was more frequent (100%) than among those that did not develop aspergillosis (35 out of 55; 63.6%) (*p* = 0.012), as well as GOLD IV patients were more frequent among COPD patients developing aspergillosis than among COPD patients that did not (50.0 vs. 26.1%, *p* = 0.046). Mean time to development of aspergillosis was 18.4 months (median: 8.5) with a wide range (1–58). Overtime, the percentage of patients developing aspergillosis was significantly higher among GOLD IV patients than among GOLD II-III patients (*p* = 0.032).

**Conclusions:**

The high percentage of cases progressing to aspergillosis among colonized patients, especially among those with COPD (25.5%), stresses the importance of colonization as risk factor, and creates awareness of the possible change from colonization to invasive disease in GOLD IV patients.

## Background

The significance of *Aspergillus* colonization is unclear because it may represent a temporary passage, a long-term benign carriage or a sign preceding invasive disease, with scarce information on the length of the incubation period [1, 2]. A previous study by our group explored implications of repeated isolation of *Aspergillus* spp. from lower respiratory samples in non-transplant, non-neutropenic patients in an attempt to identify factors helping in discriminating colonization and true infection [3]. One important finding was the high number of patients with probable/proven aspergillosis among patients with chronic obstructive pulmonary disease (COPD) [3], including GOLD (Global Initiative for Chronic Obstructive Lung Disease) II patients [4].

The cohort of patients categorized as colonized in the previous study offered the opportunity to explore the possible progression and time to development of aspergillosis in non-transplant, non-neutropenic patients as previously explored for post-lung transplant patients [5] and patients with acute myeloid leukemia [6].

The aim of the present follow-up study was to explore the evolution of colonized patients from the cohort identified in the previous study [3] to determine the percentage of cases progressing to aspergillosis and time to development.

## Methods

A retrospective review of clinical records of patients categorized as *Aspergillus*-colonized patients in a previous study [3, 4] was performed. The inclusion criterion was *Aspergillus*-colonized patients defined as patients with at least two cultures of lower respiratory samples (good quality sputum) yielding *Aspergillus* spp. without dyspnoea exacerbation, bronchospasm, new pulmonary infiltrates or symptoms attributable to *Aspergillus* in the previous study. A total of 106 colonized patients were identified in the database of the previous study, 93 of them surviving at the time of study closure [3]. Centres (22 centres) that attended these 93 patients were contacted and invited to participate in the present follow-up, and 17 of them agreed. In these participating centres, a total of 67 colonized patients were followed-up. Clinical records were retrospectively reviewed, and clinical data from all medical visits/hospital admissions due to respiratory symptoms suffered by these patients (whether as outpatients or inpatients, and regardless the aetiology of infections) from the end date of the past study (individualized for each patient) to April 2015 were recorded. The study protocol was approved by the Ethics Committee of Hospital Universitario Monteprincipe, Madrid, Spain.

Demographic data, underlying illnesses, clinical and radiological data, laboratory data, previous treatments (corticosteroids, antibiotics, antifungals…), antifungal treatment and outcome were recorded. The Charlson comorbidity index and its correction by age [7, 8], the modified McCabe score (Sabadell score) [9], and the Acute Physiologic and Chronic Health Evaluation (APACHE) II score were retrospectively calculated, as well as the GOLD classification for COPD patients [10]. When more than one respiratory episode was recorded during the follow-up period, data from the episode where aspergillosis was diagnosed or data corresponding to the last episode (for patients not developing aspergillosis) were considered.

Diagnoses of tracheobronchitis were always based on bronchoscopy reports included in clinical records. Simple tracheobronchitis was considered when the bronchoscopy report described mucosal inflammation and mucus secretions, and invasive tracheobronchitis when ulceration and pseudomembrane formation was observed [3, 11]. Diagnosis of invasive pulmonary aspergillosis was based on a host factor criterion (COPD), clinical criteria (dyspnoea exacerbation, bronchospasm, new pulmonary infiltrates or other new respiratory symptoms…), and mycological criteria [2]. Chronic pulmonary aspergillosis was considered when long-term fibrotic lesions with or without necrosis or cavitations had been recorded in the patient’s clinical records [3, 12]. Aspergilloma was considered on a radiological basis (upper-lobe, mobile, intracavitary mass) [12, 13]. Allergic bronchopulmonary aspergillosis (ABPA) was considered in patients with asthma/cystic fibrosis based on clinical data (worsening or appearance of new respiratory symptoms), analytical data (including specific immunoglobulins) and radiological data [14].

Comparisons of variables between colonized patients that developed aspergillosis and those that did not were performed by the χ^2^ test and the Fisher’s exact test, when necessary. For quantitative variables, since data did not show normality in the Kolmogorov – Smirnoff test, the Kruskal-Wallis and Mann-Whitney tests, when necessary, were used. For each patient developing aspergillosis, time from colonization to infection was calculated considering the date of his first positive *Aspergillus* culture (in the previous study) and the visit/admission date (in the present follow-up) when the diagnosis of aspergillosis was made. A Kaplan-Meier curve was used to describe time to development of aspergillosis. Statistical analyses were performed using SPSS v 14 programme (SPSS Inc., Chicago IL).

## Results

Twelve of the 67 colonized patients (17.9%) included in this follow-up study developed aspergillosis. Table 1 shows characteristics of patients distributed by development of aspergillosis or not. During the study period, patients suffered several episodes of respiratory infections, the number of episodes being higher among patients developing aspergillosis, although the difference did not reach significance. No differences between groups were found in values of clinical index/scores or ICU admission. Forty-seven of the 67 (70.4%) colonized patients presented COPD: all patients developing aspergillosis were COPD patients and 35 out of 55 (63.6%) of those not developing aspergillosis presented COPD, the difference being statistically significant (*p* = 0.012). Colonising species were identified as *Aspergillus fumigatus* in 46 (68.7%) patients, *Aspergillus niger* in three (4.5%) and *Aspergillus flavus* in two (3.0%) patients, the remaining being reported as *Aspergillus* spp. Non-*fumigatus* species were isolated from 2/12 (16.7%) patients developing aspergillosis and 19/55 (34.5%) from those that did not (*p* = 0.313).

**Table 1:** Characteristics of patients distributed by development or not of aspergillosis

	Total	Developing aspergillosis		*p*
		No	Yes	
n	67	55	12	
No. respiratory episodes; median (IQR)	2 (1.0–4.0)	2 (1.0–3.0)	3 (2.0–4.8)	0.061
Age; mean ± SD	73.1 ± 14.9	73.7 ± 15.4	70.2 ± 12.7	0.468
≥70 years	48 (71.6)	49 (72.7)	8 (66.7)	0.729
COPD	47 (70.4)	35 (63.6)	12 (100)	0.012
GOLD category				
III	17 (29.3)	14 (30.4)	3 (25.0)	1.000
IV	18 (31.0)	12 (26.1)	6 (50.0)	0.046
Diabetes mellitus II	13 (19.4)	11 (20.0)	2 (16.7)	1.000
Congestive heart failure	9 (13.4)	7 (12.7)	2 (16.7)	0.658
Liver disease	7 (10.4)	5 (9.1)	2 (16.7)	0.600
Chronic renal insufficiency	7 (10.4)	7 (12.7)	0 (0.0)	0.336
Charlson index; mean ± SD	2.4 ± 1.7	2.4 ± 1.8	2.6 ± 1.6	0.744
APACHE; mean ± SD	12.5 ± 5.0	12.1 ± 5.0	14.4 ± 5.0	0.142
McCabe (rapidly fatal)	11 (16.4)	9 (16.3)	2 (16.7)	1.000
ICU admission	5 (7.5)	4 (7.3)	1 (8.3)	1.000
Mechanical ventilation	6 (9.0)	5 (9.1)	1 (8.3)	1.000
Previous steroids intake (3 months)	24 (35.8)	20 (36.4)	4 (33.3)	1.000
Previous antibiotics intake (30 days)	28 (41.8)	22 (40.0)	6 (50.0)	0.538
Previous antifungals intake (30 days)	9 (13.4)	7 (12.7)	2 (16.7)	0.658
Antifungals in the present episode	7 (10.4)	2 (3.6)	5 (41.7)	0.001

For patients without aspergillosis, data correspond to episodes with the highest GOLD/APACHE value. Data are expressed as *n* (%) except where indicated

CT scan was performed in 29.9% patients: 50% of patients developing aspergillosis and 25.5% of those that did not (*p* = 0.160).

No differences between groups were found in the number of patients receiving antibiotics or antifungals prior to the described episode, with quinolones and voriconazole as most frequent compounds. Antifungal treatment was more frequently administered during the described episode in patients developing aspergillosis (41.7 vs. 3.6%, *p* = 0.001), being voriconazole the most frequent compound [4 out of 5 (80%) treated-patients developing aspergillosis and 1 out 2 (50%) among those treated-patients that did not]. With respect to previous intake of steroids, doses >20 mg/day had been taken by 3 out of 12 (25.0%) patients developing aspergillosis and 14 out of 55 (25.5%) patients that did not (*p* = 1.000), with 0% and 12.7% (7/55) patients, respectively, having received cumulative doses >700 mg within the previous three months (*p* = 0.355). Although non-significant, the percentage of patients developing aspergillosis treated with cumulative doses of 100-700 mg within the previous three months was higher than among those not developing aspergillosis (5 out of 12–41.7%- vs. 11 out of 55–20%-; *p* = 0.140).

In the subgroup of 47 COPD patients, the percentage of patients developing aspergillosis was 25.5% (12 out of 47), being the percentage of GOLD IV patients significantly higher among patients developing aspergillosis than among those that did not develop aspergillosis (50.0% vs. 26.1%, *p* = 0.046).

**Fig. 1:**
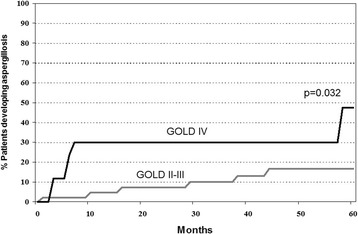
Overtime percentage of GOLD II-III and GOLD IV patients developing aspergillosis among the 47 COPD patients

Table 2 details data from patients developing aspergillosis. Most patients were males (75%) and ≥65 years old (83.3%). Diagnoses included six tracheobronchitis (4 invasive and 2 simple tracheobronchitis), four pulmonary disease (2 invasive pulmonary aspergillosis and 2 chronic pulmonary aspergillosis), one ABPA and one pulmonary aspergilloma. Presence of *A. fumigatus* in lower respiratory tract samples were confirmed at the time of diagnosis (eight patients) or within the previous two months in 10 out of 12 patients with aspergillosis, lacking recent positive culture for an 80-years old GOLD IV patient with invasive tracheobronchitis and the patient with aspergilloma. CT scans performed in six patients showed infiltrates (five patients), nodules (three patients), cavitations (one patient) and pleural effusion (one patient), without halo signs or air crescent signs. No samples for histopathological confirmation were collected in these patients. Mean time to development of aspergillosis was 18.4 months (median: 8.5 months), with a wide range (1–58 months). Figure 1 shows overtime the percentage of patients developing aspergillosis among GOLD II-III patients and GOLD IV patients. Significant differences were found between both groups (*p* = 0.032).

**Table 2:** Detailed data from patients with aspergillosis

Patient no.	Gender	Age	GOLD	Time to aspergillosis (months)	Diagnosis
1	Male	70–75	IV	6	Aspergilloma
2	Female	<50	II	16	ABPA
3	Male	60–69	IV	7	CPA
4	Male	>75	II	38	CPA
5	Female	>75	III	29	IPA
6	Male	70–75	IV	3	IPA
7	Male	>75	IV	58	IT
8	Male	>75	II	44	IT
9	Male	60–69	III	10	IT
10	Female	>75	IV	6	IT
11	Male	>75	IV	3	ST
12	Male	60–69	III	1	ST

*ABPA* Allergic bronchopulmonary aspergillosis; *CPA* Chronic pulmonary aspergillosis; *IPA* Invasive pulmonary aspergillosis; *IT* Invasive tracheobronchitis; *ST* Simple tracheobronchitis

## Discussion

Nowadays, approximately 30–50% cases of invasive pulmonary aspergillosis are diagnosed in non-neutropenic patients [15], and pre-existing structural disease of the lungs such as COPD increases the risk for developing invasive pulmonary aspergillosis [16]. A previous study in our country reported 22.1% of probable invasive pulmonary aspergillosis among hospitalized COPD patients with at least one culture positive to *Aspergillus* [17]. Although several factors (among them, previous colonization, not sufficiently studied) have been identified, no definitive data is available to explain why colonization changes to invasive disease [18] with some patients with COPD developing invasive pulmonary aspergillosis while others do not [15]. The present study, a follow-up study in patients with *Aspergillus* airway colonization, showed that approximately 18% patients developed aspergillosis, this percentage being higher among patients with COPD (approx. 26%). In the present series, COPD was the sole risk factor in common for our non-transplant, non-neutropenic patients developing aspergillosis, 75% of them presenting GOLD III-IV. Other conditions compromising immune function and reported to increase the risk of acquiring invasive aspergillosis as diabetes mellitus or liver cirrhosis [16] were present in some of the patients developing aspergillosis, but no conclusions can be drawn in our study on colonized patients. Similarly, previous intake of antibiotics and steroids has also been reported as an increased risk for invasive disease [19], but in the present series the percentage of patients that had been treated with antibiotics or high doses of steroids prior to the index episode was similar in both groups, as there are common drugs for patients with COPD exacerbations.

Once tissue invasion has occurred, it is well known that *Aspergillus* produces a wide range of invasive and saprophytic syndromes [1], *A. fumigatus* being the main responsible species. This range of diseases was also found in the present study where two patients developed a saprophytic syndrome (aspergilloma and ABPA) and, more important from the prognosis perspective, 50% of patients presented an invasive syndrome. The high mortality rate associated with invasive disease in non-neutropenic critically ill patients has been attributed to difficulties in timely diagnosis due to insensitive and non-specific clinical signs and lack of unequivocal diagnosis criteria [20]. Diagnosis presents special difficulties in COPD patients since the invasiveness of procedures to obtain samples for histopathological confirmation limits the possibility of fungal evidence because these procedures are rarely performed in patients suffering from chronic lung disease as late-stage COPD patients [17, 21]. Therefore, most cases reported in COPD patients are “probable” [21] as occurred in all cases in the present series. In this sense, the difficulty in sample collection in elderly late-stage COPD patients was probably the reason for the lack of a recent positive culture in the 80-years old GOLD IV patient with invasive tracheobronchitis in the present study. However, CT scan and antifungal therapy were markedly more frequent in patients developing aspergillosis than in those that did not. CT scan is indicated in cases of suspicion of *Aspergillus* involvement, regardless classical radiological findings may be less reliable in non-neutropenic patients [16], as in the present series where none of the patients presented halo or air crescent signs.

To our knowledge, this is the first follow-up study exploring the percentage of colonized patients developing aspergillosis among non-transplant, non-neutropenic patients. Our results showed a percentage of development among COPD patients (25.5%) similar to that reported in pre-transplant colonized cystic fibrosis patients developing invasive aspergillosis after transplantation (24.6%) [5]. In cystic fibrosis patients, *Aspergillus* colonization is associated with pulmonary exacerbations, and a small subset of patients develops allergic aspergillosis and very rarely invasive aspergillosis [5]. However, after transplantation *Aspergillus* should not be considered so innocuous since the impaired immunity precipitates invasive syndromes [5].

Our cohort of colonized patients was retrospectively followed to determine not only the percentage of aspergillosis development but also time to development. The duration of the incubation period of invasive aspergillosis remains unknown, probably due to the lack of knowledge regarding time of exposure, duration of previous colonization and the relationship between exposure, colonization and invasion [6]. Considering the lack of specific studies in this field, in the commented study on colonized cystic fibrosis patients, median time from transplantation to infection was 42 days [5]. Time from colonization to infection in our study could be hardly compared with the reported time from transplantation to infection since post-transplant patients receive immunosuppressive agents that precipitates an impaired immunity, making the great difference with COPD patients. Thus, colonization, percentage of patients developing aspergillosis and time to aspergillosis should be necessarily different in COPD patients. In this sense, the lower percentage of patients developing invasive syndromes (6 out of 47, 12.8%) and the larger time to develop aspergillosis (8.5 months median time) among COPD patients in the present study could be related to the absence of induced impair immunity by immunosuppressive agents.

Although this is the largest series of colonized non-transplant, non-neutropenic patients reporting development of aspergillosis, the low number of patients and the retrospective nature of the study (patients managed following daily practice without additional investigational tests) limit the conclusions. The fact that diagnostic criteria were based on those by Bulpa et al. [2] and not on EORTC/MSG definitions [22] could also be considered a potential study limitation, but criteria by Bulpa et al. are commonly used for non-hematological, non-transplant, non-neutropenic patients [17, 23] since EORTC/MSG definitions do not include COPD as host criteria. However, the percentage of patients developing aspergillosis among colonized COPD patients and the multicentre nature of the study deserves attention to study conclusions, regardless the potential variability in daily practice between centres.

## Conclusions

The high percentage of cases progressing to aspergillosis among *Aspergillus*-colonized non-transplant, non-neutropenic patients, especially among patients with COPD, stresses the importance of colonization as risk factor, and creates awareness of the possible change from colonization to invasive disease in GOLD IV patients.

## Abbreviations

APACHE II: Acute Physiologic and Chronic Health Evaluation
COPD: Chronic obstructive pulmonary disease
GOLD: Global Initiative for Chronic Obstructive Lung Disease
